# Identification of Fatty Acids, Amides and Cinnamic Acid Derivatives in Supercritical-CO_2_ Extracts of *Cinnamomum tamala* Leaves Using UPLC-Q-TOF-MS^E^ Combined with Chemometrics

**DOI:** 10.3390/molecules29163760

**Published:** 2024-08-08

**Authors:** Hema Lohani, Arvind Kumar, Vinod Bidarakundi, Lalit Agrawal, Syed Zafar Haider, Nirpendra Kumar Chauhan

**Affiliations:** Centre for Aromatic Plants (CAP), Industrial Estate, Selaqui, Dehradun 248011, India; hemalohani2004@rediffmail.com (H.L.); bidarkundivinod193@gmail.com (V.B.); lalit.ncpgr@gmail.com (L.A.); zafarhrdi@gmail.com (S.Z.H.)

**Keywords:** *Cinnamomum tamala*, chemometrics, fatty acids, fatty acid amides, SC-CO_2_ extraction, UPLC-Q-TOF-MS^E^

## Abstract

*Cinnamomum tamala* leaf (CTL), also known as Indian bay leaf, is used all over the world for seasoning, flavoring, and medicinal purposes. These characteristics could be explained by the presence of several essential bioactive substances and lipid derivatives. In this work, rapid screening and identification of the chemical compounds in supercritical (SC)-CO_2_ extracts of CTL by use of UPLC-Q-TOF-MSE with a multivariate statistical analysis approach was established in both negative and positive mode. A total of 166 metabolites, including 66 monocarboxylic fatty acids, 52 dicarboxylic fatty acids, 27 fatty acid amides, and 21 cinnamic acid derivatives, were tentatively identified based on accurate mass and the mass spectrometric fragmentation pattern, out of which 142 compounds were common in all SC-CO_2_ extracts of CTL. Further, PCA and cluster hierarchical analysis clearly discriminated the chemical profile of analyzed extracts and allowed the selection of SC-CO_2_ extract rich in fatty acids, fatty acid amides, and other bioactive constituents. The result showed that the higher number of compounds was detected in CTL4 (300 bar/55 °C) extract than the other CTL extracts. The mono- and di-carboxylic fatty acids, fatty acid amides, and cinnamic acid derivatives were identified in CTL for the first time. UPLC-Q-TOF-MS^E^ combined with chemometric analysis is a powerful method to rapidly screen the metabolite profiling to justify the quality of CTL as a flavoring agent and in functional foods.

## 1. Introduction

*Cinnamomum* (Lauraceae) is a genus comprising over 250 species of evergreen trees found in sub-tropical and tropical Asia, Africa, and South America, valued for their culinary and medicinal uses [1]. Among them, *Cinnamomum tamala* (Buch.-Ham.) T. Nees & Eberm., commonly known as Tejpat, Indian cassia, or Indian bay leaf, is one of the most commercially important species of the genus [2]. This species is naturally distributed in the North-East Himalayas, North-Western Himalayas, and southern parts of the country from tropical to sub-tropical regions at altitudes of 900–2500 m [3,4]. The leaves and bark of *Cinnamomum trees* are widely utilized as spices in cooking and for producing essential oils and have many applications in perfumery, flavoring, and pharmaceuticals industries [5]. *Cinnamomum tamala* leaves (CTLs) are most popular as a food additive in numerous culinary preparations worldwide. In India, CTLs are used not only as spices and flavoring agents but also for their medicinal properties, addressing conditions such as diabetes, hyperlipidemia, inflammation, hepatotoxicity, and diarrhea [6]. Since ancient times, CTLs have been traditionally utilized in Ayurvedic and Unani medicine to treat conditions related to scabies, the anus, rectum, liver, and spleen [7].

Moreover, research into the pharmacological activities of *C. tamala* has highlighted its various benefits, including antimicrobial, antioxidant, anti-inflammatory, analgesic, antiulcerogenic, antihypertensive, antidiabetic, antidiarrheal, antipyretic, anti-obesity, cardiovascular protective, and neuroprotective effects [3,6,8,9]. Phytochemical studies of *C. tamala* extracts have identified several bioactive compounds such as terpenes, alkaloids, flavonoids, tannins, polyphenols, saponins, and fatty acids [10,11,12,13,14]. Among these, fatty acids (FAs) are particularly notable due to their significant biological functions and health benefits, including roles in lipid metabolism, antioxidation, anti-inflammation, cholesterol lowering, and augmenting the liver detoxification process [15,16,17,18,19]. For instance, linoleic and linolenic acids have been reported to offer protective effects against cardiovascular diseases, inflammatory conditions, and neurodegenerative disorders like Alzheimer’s disease. When fatty acids combine with amines, they form fatty acid amides (FAAs), which have varying carbon lengths and unsaturation. These bioactive intracellular signaling molecules are regulated by fatty acid amide hydrolases, which convert FAAs back into their parent fatty acids [20,21]. Despite their importance, there is limited research on the fatty acids in *C. tamala*. The study by Farag et al. in 2022 is the only one focusing on identifying fatty acids in *C. tamala* bark [12]. Owing to the high medicinal value and effects of these components, it is crucial to characterize the fatty acids and FAAs in CTLs and develop an efficient green extraction method to minimize postprocessing requirements.

For this purpose, supercritical carbon dioxide (SC-CO_2_) is a green extraction technique that has gained attraction as an alternative to traditional methods for extracting fatty acids [22,23]. SC-CO_2_ has advantages such as nontoxicity, selectivity, absence of solvent residues, and operation at low temperatures, making it suitable for extracting hydrophobic compounds without degrading active metabolites. Mass spectrometry (MS) has been extensively employed for the analysis of fatty acids, fatty acid amides, and fatty acid derivatives in targeted samples. Gas chromatography coupled with EI-MS is generally applied to analyze the fatty acids by derivatization to their respective fatty acid methyl esters (FAMEs) [24]. In addition, liquid chromatography (LC)-MS is an effective tool for fatty acid analysis due to its high sensitivity, selectivity, and rapid analysis capabilities [25], and it also screens the chemical constituents in herbal extracts even at the sub ppm level [26]. Q-TOF coupled with UPLC provides not only conventional MS and MS/MS data but also gives MS^E^ for comprehensive accurate mass precursor and fragment ion information [27]. This method can be used to consecutively scan by “low collision energy” and “high collision energy” in two channels, which provide the highly accurate information of parent ions and fragment ions within a single analysis.

This study aims to optimize the extraction conditions by investigating the metabolite profile of CTL extracts prepared by SC-CO_2_ technique. A UPLC-Q-TOF-MS^E^ technique combined with a chemometric approach will be used for the first time to rapidly screen and identify the fatty acids, fatty acid amides, and cinnamic acid derivatives in various different SC-CO_2_ extracts of CTL.

## 2. Results and Discussion

### 2.1. Extraction Yield

Exhaustive drying experiments (110 °C, continued until no weight decrease was registered) showed that the average moisture content was 6.3 ± 0.28% of the shade-dried *C. tamala* leaves (CTL) powder. For efficient and appropriate SC-CO_2_ extraction, the optimized parameters, i.e., temperatures (55 °C), desired pressure (100, 150, 250, 300, and 500 bar), particle diameter (<1.0 mm), and tested extraction time (3 h), were applied in triplicate for each set of experiments. The extraction yields (%) of CTL extracts were 0.48 ± 0.04% at 100 bar/55 °C, 3.41 ± 0.56% at 150 bar/55 °C, 3.93 ± 0.01% at 250 bar/55 °C, 4.87 ± 0.54% at 300 bar/55 °C, and 7.94 ± 0.02% at 500 bar/55 °C, respectively.

### 2.2. UPLC-Q-TOF-MS^E^ Analysis and Metabiltes Identification

**Figure 1:**
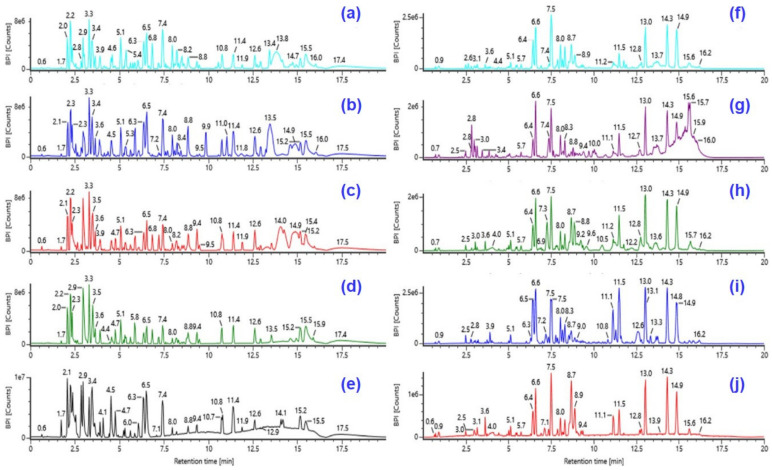
Base peak chromatograms (BPCs) of *C. tamala* leaf SC-CO_2_ extracts: (**a**) CTL1, (**b**) CTL2, (**c**) CTL3, (**d**) CTL4, and (**e**) CTL5 in positive ESI; (**f**) CTL1, (**g**) CTL2, (**h**) CTL3, (**i**) CTL4, and (**j**) CTL5 in negative ESI modes.

Optimized chromatographic and mass spectral analysis were performed to characterize the bioactive compounds in the SC-CO_2_ extracts of CTL. Each extract (1.0 mg/mL, ca. 1000 ppm) solution was prepared using HPLC analytical-grade solvent MeOH, filtered with a membrane disc filter, and then subjected to UPLC-Q-TOF-MS analysis. Isocratic and gradient UPLC methods were tested to optimize the conditions for maximum resolution of peaks. Different mobile phases (water/acetonitrile, 0.1% formic acid in water/acetonitrile, water/methanol, and 0.1% formic acid in water/methanol) at variable flow rates (0.25, 0.3, 0.4, and 0.5 mL/min) were examined and compared for better chromatographic separation and appropriate ionization. A mobile phase consisting of 0.1% aqueous formic acid and acetonitrile at a flow rate of 0.3 mL/min resulted in satisfactory separation in a short analysis time. CTL extracts were analyzed in the negative ionization modes using a Xevo G2-XS mass spectrometer, and the base peak chromatograms (BPCs) are shown in Figure 1. Due to the complexity of chemical composition in herbal extracts, we established a post-targeted screening strategy for the identification of lipids in different SC-CO_2_ extracts of CTL. The accurate masses of targeted [M + H]^+^ and/or [M − H]^−^ ions of all possible fatty acids and fatty acid amides were extracted at the Waters Connect UNIFI workstation using a mass tolerance window of ±7 ppm, and the respective peak retention times (RT) are reported in Table 1. The mass spectra derived from these extracted ion chromatograms (EICs) show intense [M + H]^+^ and/or [M − H]^−^ ions with a mass error ≤ 6.5 ppm. The expected compound showed distinguishable MS/MS characteristic fragment ions with high mass accuracy. Compounds were tentatively identified by determining the elemental compositions of the precursor and product ions. The molecular formula and rational fragmentation patterns and pathways of these compounds were then identified based on a comparison of these data with chemical compound databases. In this way, we used the UPLC-Q-TOF-MS^E^ method in combination with databases to screen 166 compounds from CTL extracts.

#### 2.2.1. Identification of Fatty Acids

**Figure 2:**
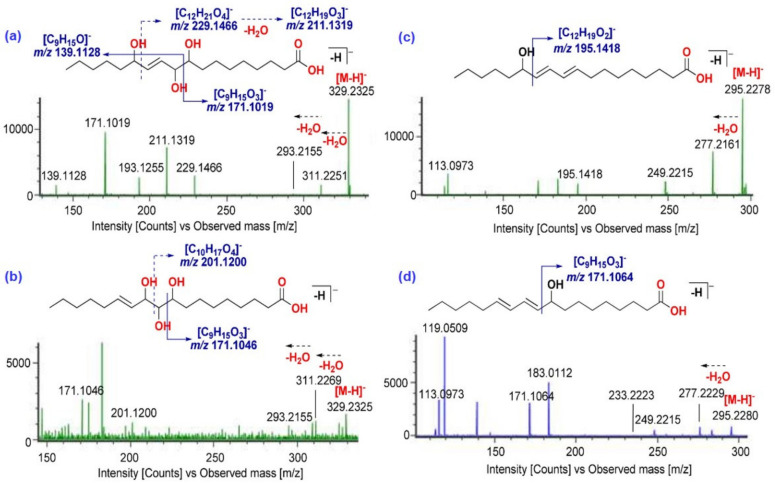
MS/MS spectra of hydroxyl derivatives of monocarboxylic fatty acids: (**a**) 9,10,13-trihydroxy-11-octadenoic acid, (**b**) 9,10,11-trihydroxy-12-octadenoic acid, (**c**) 13-hydroxy-9,11-octadecadienoic acid, and (**d**) 9-hydroxy-10,12-octadecadienoic acid.

FAs are a group of chemical compounds that contain a carboxylic acid functional group (–COOH) at one end of their hydrocarbon chain. In this study, two types of FAs were detected, with one being a monocarboxylic FA containing one –COOH group, while the second one was a dicarboxylic FA, containing two –COOH groups [28]. A total of 66 peaks have been extracted from TICs and tentatively identified as monocarboxylic FAs. A total of 19 peaks out of 66 have been observed to be saturated monocarboxylic FA, as they contain no double bonds in their carbon chain, based on their HRMS, empirical formula, and double bond equivalents (DBE). Saturated FAs showed a positive relationship between retention time and the length of FA, which indicates that the elusion time increases as the carbon length of fatty acid increases. Also, they showed strong [M − H]^−^ ion in both channels, i.e., low-energy CID and high-energy CID. On the other hand, the lack of detection of fragment ions of the linear hydrocarbon backbone is in accordance with the previous reports [29]. In the high-energy CID channel, the [M − H]^−^ ion did not lead to a decrease when using the highest energy in MS^E^ experiment up to 85 eV. They showed characterization ions corresponding to [M − H − 18]^−^, [M − H − 46]^−^, and [M − H − 44]^−^ ions, resulting from a loss of one water molecule, loss of -HCOOH, and decarboxylation from quasimolecular ions, respectively (Figure 2). Eleven peaks at 66, 67, 74, 76, 106, 108, 112, 129, 146, 153, and 158 have been detected as the most abundant monocarboxylic FAs in five different SC-CO_2_ CTL extracts and tentatively identified as 9-hydroxy-12,14,16-octadecatrienoic acid (*t_R_* = 6.42 min), hydroxyoctadecatrienoic acid (*t_R_* = 6.57 min), 13-hydroxy-9,11-octadecadienoic acid (*t_R_* = 7.49 min), hydroxyoctadecatrienoic acid II (*t_R_* = 8.30 min), oleic acid (*t_R_* = 11.14 min), stearic acid I (*t_R_* = 11.24 min), linolenic acid (*t_R_* = 11.48 min), linoleic acid (*t_R_* = 13.04 min), palmitic acid III (*t_R_* = 14.30 min), ocatdecanoic acid II (*t_R_* = 14.87 min), tetracosanoic acid (*t_R_* = 15.50 min), and docosanoic acid or behenic acid (*t_R_* = 15.70 min), respectively, based on exact mass and MS/MS data [30]. Monocarboxylic FAs have been detected as the most abundant in CTL4 (300 bar/55 °C) SC-CO_2_ extract compared to other extracts.

Similarly, total 52 dicarboxylic fatty acids have been tentatively identified in CTL extracts. Monitoring the high-energy CID channel, fragment spectra revealed no fragmentation for many fatty acids, while the formation of [M − H − 18]^−^, [M − H − 44]^−^, and [M − H − 18 − 44]^−^ ions was observed in low intensity, resulting from a loss of water molecules, decarboxylation, and simultaneous loss of water and CO_2_ molecules, respectively (Table 1 and Figure 2). Around 28 peaks out of 52 have been tentatively identified as saturated dicarboxylic fatty acids having a carbon chain length of 7 to 25. The [M − H]^−^ ion of 13 peaks was tentatively identified as unsaturated dicarboxylic FA having one unsaturation, while three peaks at 105 (*t_R_* = 11.10 min), 117 (*t_R_* = 11.88 min), and 120 (*t_R_* = 12.10 min) has two unsaturations. Moreover, eight peaks have been identified as oxygenated dicarboxylic FA based on their exact mass, empirical formula, DBE, characteristic fragment ions, and literature support. Peaks 15, 55, 56, 57, 59, 107, 119, 130, 148, 149, and 163 have been identified as the most abundant peaks corresponding to azelaic acid (*m*/*z* 187.0982), octadecenedioic acid I (*m*/*z* 311.2224), octadecanedioic acid (*m*/*z* 313.2375), octadecenedioic acid II (*m*/*z* 311.2224), octadecenedioic acid III (*m*/*z* 311.2224), pentacosanedioic acid (*m*/*z* 411.3474), heneicosanedioic acid (*m*/*z* 355.2850), docosanedioic acid (*m*/*z* 369.3010), tricosanedioic acid (*m*/*z* 383.3176), octadecanedioic acid VI (*m*/*z* 313.2375), and tetracosanedioic acid (*m*/*z* 397.3307), respectively. Trihydroxyoctadecanoic acid showed main MS/MS fragments (Figure 2) at *m*/*z* 311 and 293 due to subsequent loss of two water molecules and a main fragment at *m*/*z* 211 due to C15\C16 bond cleavage [31]. Dicarboxylic FAs have also been detected at maximum intensity in CTL4 (300 bar/55 °C) SC-CO_2_ extract. Recently, Farag et al., in their 2022 study, have reported several fatty acids (mono- and di-carboxylated) from the bark of different cinnamon species, including *C. tamala* [12]. To the best of our knowledge, there is no report on the identification of fatty acids in CTL.

#### 2.2.2. Identification of Fatty Acid Amides

**Figure 3:**
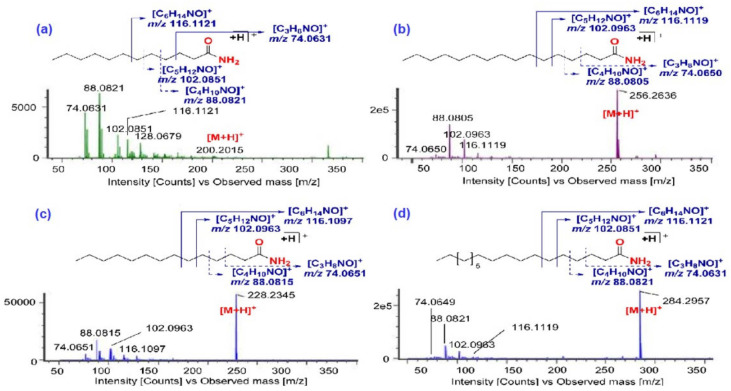
MS/MS spectra of fatty acid amides: (**a**) lauramide, (**b**) palmitamide, (**c**) myristamide, and (**d**) stearamide.

Generally, FAAs are bioactive lipid signaling molecules that play key roles in biological activities such as analgesic, antianxiety, anti-convulsion, anti-epilepsy, neuroprotection, and weight loss functions. In our study, 27 peaks were observed as the [M + H]^+^ ion in positive ion mode (ESI+) and their empirical formula assigned to C, H, O, and single N atoms that are present in the structure. Out of these, 16 peaks were tentatively identified as saturated FAAs based on their exact mass, empirical formula, and one double bond equivalent (DBE) and they were similar regardless of the acyl chain length ranging from C_9_ to C_22_. Also, they were discovered to have similar fragment ion peaks containing carbon, hydrogen, oxygen, and nitrogen, which were fragments having the amide head group with variation in the acyl fragmentation site. The MS/MS spectra of the [M + H]^+^ ion of these peaks showed the fragment ions at the *m*/*z* 116.1123 [C_6_H_14_NO]^+^, *m*/*z* 102.0897 [C_5_H_12_NO]^+^, *m*/*z* 88.0739 [C_4_H_10_NO]^+^, and *m*/*z* 74.0631 [C_3_H_8_NO]^+^ corresponding to the cleavage of acyl chain (Figure 3); accordingly, these peaks were identified as lauramide (*t_R_* = 6.40 min), palmitamide (*t_R_* = 11.80 min), myristamide (*t_R_* = 9.29 min), and stearamide (*t_R_* = 14.65 min), respectively [25,32]. The empirical formula of the [M + H]^+^ ion of eight peaks (83, 123, 134, 136, 137, 143, 151, and 157) were found to be two double bond equivalents (DBE), one corresponding to an amide group and one corresponding to unsaturation in the acyl chain. The MS/MS spectra of these compounds showed fragments corresponding to the cleavage of the acyl fragmentation site. Palmitoleamide (C16:1, *t_R_* = 9.11 min) (*m*/*z* 254.2483), heptadecenamide (C17:1, *t_R_* = 13.41 min) (*m*/*z* 268.2641), oleamide (C18:1, *t_R_* = 12.51 min) (*m*/*z* 282.2787), eicosenamide (C20:1, *t_R_* = 15.16 min) (*m*/*z* 310.3092), and erucamide (C22:1, *t_R_* = 13.51 min) (*m/z* 338.3438) were tentatively identified as monosaturated FAAs in CTL extracts based on their exact mass and literature support [30]. In addition to saturated and monosaturated FAAs, di- and trisaturated FAAs were also identified in CTL extracts based on their exact mass, empirical formula, and DBE. Peaks 100 (*t_R_* = 10.66 min) at *m*/*z* 280.2631 and 162 (*t_R_* = 15.70 min) at *m*/*z* 280.2628 were observed as [M + H]^+^ ion with empirical formula [C_18_H_34_NO]^+^ and three DBE. The MS/MS spectra of these peaks showed similar fragment ions, showing the presence of isomeric peaks. These peaks were tentatively assigned as linoleamide (C18:2) based on their fragment ion reported earlier [33]. Peak 87 (*t_R_* = 9.14 min) at *m*/*z* 278.2471, empirical formula [C_18_H_32_NO]^+^, showed four DBE (i.e., three double bonds in the acyl chain) and was tentatively assigned as linolenamide (C18:3) based on fragment ions, which were observed due to cleavages of the acyl chain. Observed molecules such as oleamide, palmitamide, and linoleamide have been reported for their hypnotic effects, analgesic effect, and potential to inhibit the migration of cancer cells, prevent Alzheimer’s disease, cardiovascular disease, inflammation, etc. [25,34,35].

#### 2.2.3. Identification of Cinnamic Acid Derivatives

**Figure 4:**
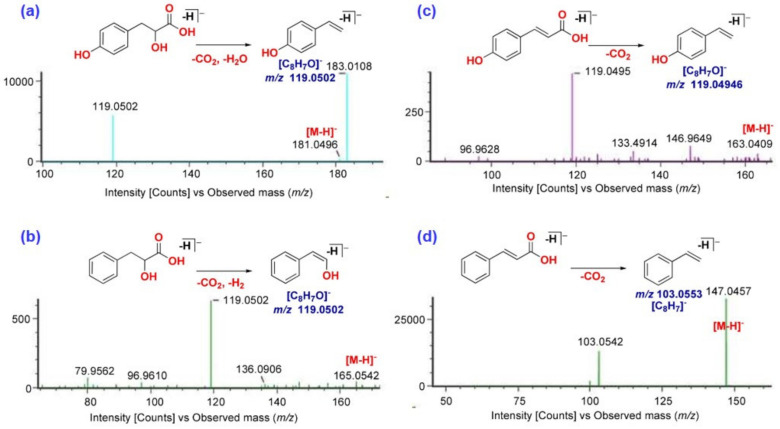
MS/MS spectra of (**a**) 3-(4-hydroxyphenyl)lactic acid, (**b**) 2-hydroxyhydrocinnamic acid, (**c**) 4-hydroxycinnamic acid, and (**d**) cinnamic acid.

Apart from FAs and FAAs, a total of 21 cinnamic acid derivatives have also been tentatively identified in CTL extracts. Out of these, 12 compounds have been tentatively identified based on their HR-MS, MS/MS, and literature support. Nine peaks out of twelve were detected as [M − H]^−^ ion in (–)-ESI, while two peaks were detected as [M + H]^+^ ion. The major identification, Peak 38 (*t_R_* = 3.21 min) with [(M + H)^+^ *m*/*z* 135.081 (C_9_H_11_O)^+^] and fragment, was observed at *m*/*z* 117.0695 [M + H − H_2_O]^+^ and identified as cinnamyl alcohol with the reference compound [12]. Peak 34 (*t_R_* = 3.06 min) at *m/z* 147.0457 was observed as [M − H]^−^ ion with empirical formula [C_9_H_8_O_2_]^−^, confirmed as cinnamic acid, which was supported by its characteristic fragment ions of *m*/*z* 103.0553 [M − H − CO_2_]^−^ (Figure 4). Peak 31 (*t_R_* = 2.94 min), 41 (*t_R_* = 3.48 min), and 53 (*t_R_* = 5.08 min) were confirmed as coumarin, *trans*-cinnamaldehyde, and *cis*-cinnamaldehyde with the reference compounds as [M + H]^+^ ion at *m*/*z* 147.0446 [C_9_H_7_O_2_]^+^, 133.0648 [C_9_H_9_O]^+^, and 133.0649 [C_9_H_9_O]^+^, respectively. Cinnamaldehyde has been reported to exhibit antibacterial, antifungal [36], antioxidant, and anti-inflammatory activities [37], including its flavor-imparting properties due to its pungent taste. Peak 31 (C_9_H_7_O_2_) obtained a quasi-ionic peak at *m*/*z* 147.0446 in ESI (+) mode, and the matching fragments were mainly *m*/*z* 103.0541 [M + H − CO_2_]^+^ and *m*/*z* 91.0540 [M + H − 2CO]^+^ (Table 1), which was consistent with the cleavage fragment of coumarin in the literature [38] and standard, so the peak was confirmed to be coumarin. These compounds, however, were detected as the major component in CTL extracts. Peak 26 (*t_R_* = 2.82 min) was detected as [M − H]^−^ ion at *m*/*z* 263.1296 [C_15_H_19_O_4_]^−^ and tentatively identified as plant hormone abscisic acid with the assistance of the library and database [12]. They were found to be most intense in CTL2 (150 bar/55 °C) SC-CO_2_ extract. Previously, various cinnamic acid derivatives, such as cinnamyl alcohol, cinnamic acid, cinnamaldehyde, and cinnamyl acetate, have been identified in *C. tamala*, which is in appropriate agreement with our finding [12,14].

### 2.3. Chemometric Analysis

**Figure 5:**
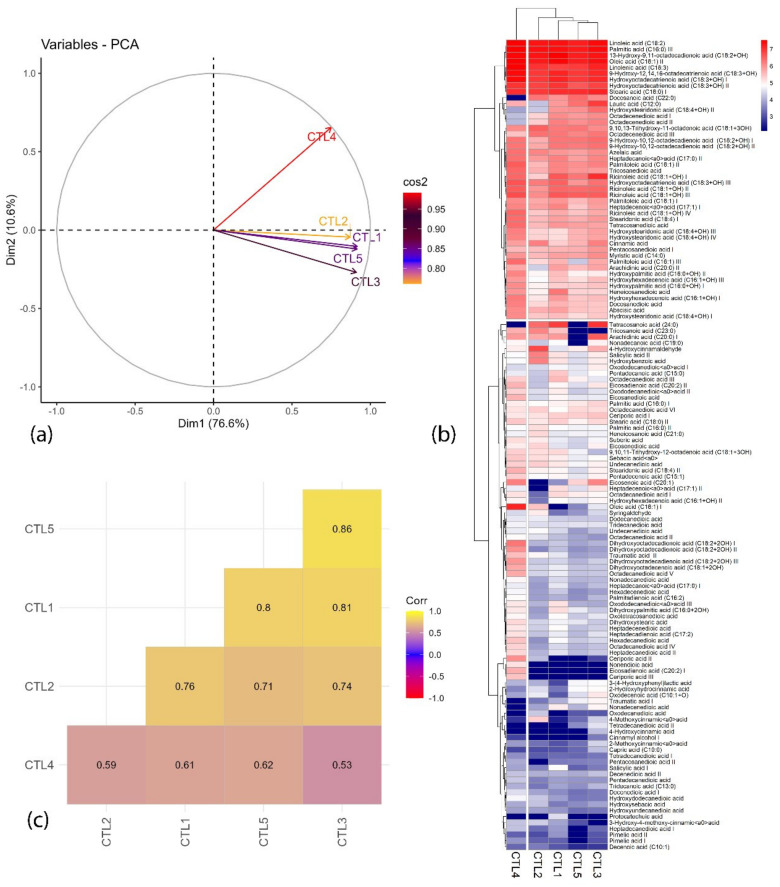
Data representing the (**a**) PCA biplot, (**b**) heatmap representing the cluster hierarchical analysis, and (**c**) correlation among different SC-CO_2_ extracts of *C. tamala* leaf in (−)-ESI mode.

**Figure 6:**
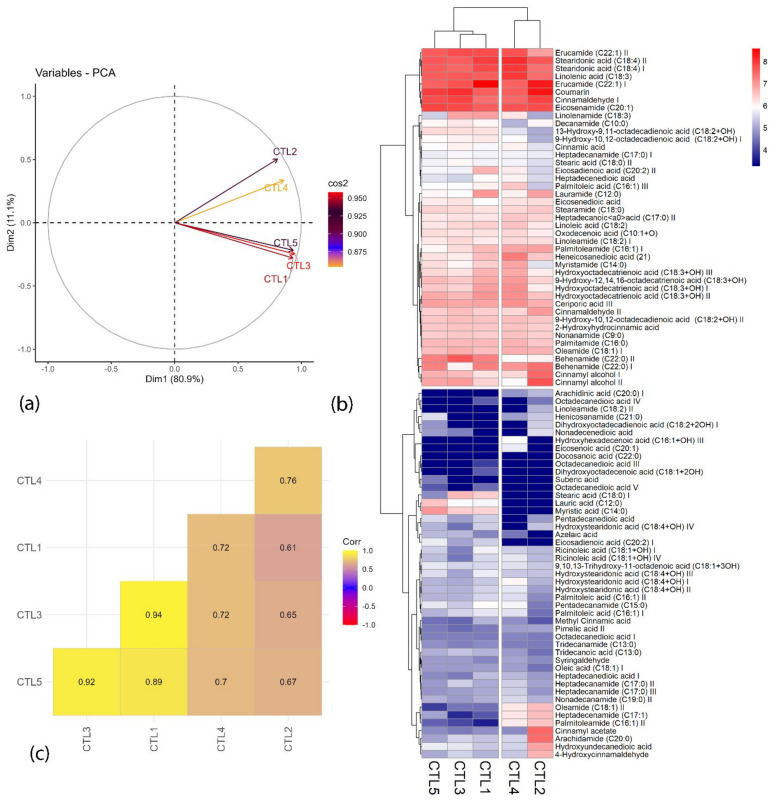
Data representing the (**a**) PCA biplot, (**b**) heatmap representing the cluster hierarchical analysis, and (**c**) correlation among different SC-CO_2_ extracts of *C. tamala* leaf in (+)-ESI mode.

Data representing the chemometric distribution of fatty acid and fatty acid amides obtained in positive and negative ionization mode in UPLC-Q-TOF-MS from the SCCO_2_ extracts at different pressures are graphically represented in Figure 5 and Figure 6. From Figure 5a and Figure 6a, it can be observed that the SC-CO_2_ extracts act differently in both modes. Two principal components (PC1 and PC2) contribute to 91.9% and 86.6% variation for both positive and negative ionization mode, respectively.

**Figure 7:**
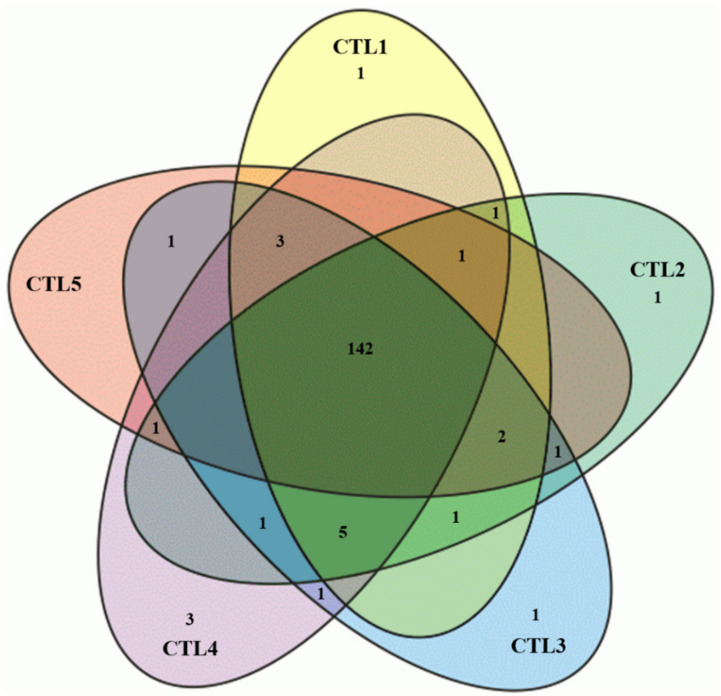
Venn diagram representing untargeted metabolites distribution in different SC-CO_2_ extracts of CTL leaves.

In negative ionization mode, among all extracts (CTL1–CTL5), CTL4 extract acts differently and contributes to the maximum variation from the other SC-CO_2_ extracts, whereas, in positive ionization mode (Figure 6a), the least variation was observed between CTL2 and CTL4, as they were clustered together and the other three extracts were clustered together. These results are supported by multivariate heatmap (Figure 5b and Figure 6b) clusters drawn based on a ward clustering method, where the rows and column are distanced apart based on the Euclidean distance. From the heatmap, it can be observed that CTL4 extract is grouped in a single separate cluster, whereas the other three extracts perform similarly and are grouped in a separate cluster. Correlation plots (Figure 5c and Figure 6c), on the other hand, exhibited a correlation between the qualitative analysis of different extracts. From Figure 6c, a good correlation (R^2^ > 0.7) can be observed between CTL3, CTL4, and CTL5, whereas a low correlation of these extracts with CTL2 and CTL4 extracts can be observed as they are separating them from each other. Conversely, for negative ESI mode, CTL4 extract behaves differently from other extracts and exhibits a low correlation (R^2^ < 0.7) with other SC-CO_2_ extracts (Figure 5c). A Venn diagram was constructed to summarize the number of metabolites that differentially accumulated in different SC-CO_2_ extracts of CTL leaves, which relatively overlap between each set of metabolites (Figure 7). A total of 166 metabolites were identified in leaves extracts; out of these, 142 metabolites were common to all five CTL extracts, projected in the center of the diagram. Notably, a highly bioactive compound known as protocatechuic acid was found exclusively in CTL1 extract (100 bar/55 °C). Protocatechuic acid has been reported to have various biological activities, for example, anti-inflammatory, neuroprotective, antiviral, anticancer, and antiaging activities [39]. It is also reported to have a protective effect against metabolic syndrome and preservation of liver, kidneys, and reproductive functions [39]. On the other hand, CTL2 (150 bar/55 °C) has linoleamide II as a fatty acid amide, which has been reported to exert sedative and hypnotic effects and inhibits the migration of cancer cells in humans [25,40]. An exclusive compound 4-hydroxycinnamic acid (HCA) was found in CTL3 (250 bar/55 °C), which is well known for its health-beneficial effects and use as cosmeceutical ingredients. HCA is mainly recognized as a potent antioxidant and is involved in the prevention of several diseases connected to oxidative stress, i.e., cardiovascular and neurodegenerative diseases and cancer [41]. Nonanedioic acid is an alpha, omega-dicarboxylic acid having a role as an antibacterial agent, an antineoplastic agent, a dermatologic drug, and a plant metabolite. Nonendioic acid, eicosadienoic acid I, and ceriporic acid III were identified in CTL4 (300 bar/55 °C). Surprisingly, CTL5 (500 bar/55 °C) extract did not have any exclusive compounds; further, it has least 151 compounds as compared to other extracts. The lower number of compounds may be due to the SC-CO_2_ extraction parameters (pressure/temperature), because high selectivity of lipophilic bioactive compounds can be easily achieved by lowering the pressure and/or temperature in the separator [42]. Based on the chemometric data, it can be observed that CTL4 extract has performed differently from the other SC-CO_2_ extracts of CTL in both ionization modes. Moreover, it could also be concluded that the SC-CO_2_ extraction parameters used in CTL4 are the optimum to achieve maximum fatty acids, fatty amides, and cinnamic acid derivatives in the present study.

## 3. Experimental

### 3.1. Chemicals and Materials

Cinnamaldehyde (93%), cinnamyl alcohol (98%), cinnamyl acetate (99%), and coumarin (99%) were purchased from Sigma Aldrich (St Louis, MO, USA). Acetonitrile and methanol (LC-MS grade) were obtained from J.T. Baker (Deventer, The Netherlands). Formic acid (LC-MS grade) was obtained from Sigma-Aldrich (St. Louis, MO, USA). Type 1 grade water, produced by Adrona Crystal, was used for all experimental procedures. High-purity gases (99.995%) for extraction were obtained from Linde (Dehradun, Uttarakhand, India).

### 3.2. Plant Materials

*C. tamala* leaves were collected from the experimental field of Centre for Aromatic Plants (CAP) under Doon Valley climatic conditions of Uttarakhand (30°36′22.13″ N, 77°84′95.38″ E) in the month of October 2021. The plant was authenticated by plant taxonomist Dr. Sunil Sah (Senior Scientist) and a voucher specimen deposited in the CAP Herbarium. Leaves were washed thoroughly with normal tap water followed by deionized water and dried at room temperature (25–30 °C). All dried leaves were crushed into coarsely ground powder (particle size < 1.0 mm, 18 mesh) using a pulverizer (Decibel, Lab Willey Grinder, Model No. DB 5581-4, New Delhi, India) and stored in an airtight container at room temperature until analysis. The moisture content of the powder was estimated to be 6.3 ± 2.8% on a dry weight basis.

### 3.3. Supercritical Fluid (CO_2_) Extraction and Sample Preparation

The coarsely ground leaves powder (2.5 kg) was charged into a 12 L extraction vessel (SS316) with a maintained constant flow rate of CO_2_ (food grade) at 0.9–1.0 kg/min (Thar SFE 2000-2-FMC50, Thar Instruments, Pittsburgh, PA, USA) for the first 15 min and the system was on a static period. After completion of the static period, the system was run at a continuous flow of CO_2_ (1.0 kg/min, 120 min), which connected to a collection chambers (separators 1 and 2), where pressure was reduced to 8.0 MPa (80 bar). The optimized extraction parameters, temperatures (55 °C), and desired pressure (100, 150, 250, 300, and 500 bar) were applied in triplicate for each set of experiments. The pressure in both the extraction and separation vessels was controlled by a pressure regulator valve. The extract in the form of oleoresin was collected from the separator and the average amount (%) of extracts was calculated. All extracts were stored in amber-colored screw-capped glass vials at 4 °C until further analysis. In total, 1.0 mg/mL solution of the dried SC-CO_2_ CTL extracts was prepared in methanol and filtered through a 0.22 µm nylon syringe filter (AXIVA Sichem Biotech, Delhi, India) prior to analysis.

### 3.4. UPLC-Q-TOF-MS^E^ Analysis

The UPLC analysis was performed on a Waters Acquity UPLC^TM^ system (Waters, Milford, MA, USA) interfaced with a Waters Xevo G2-XS Quadrupole time-of-flight mass spectrometer (Waters Corporation, Milford, MI, USA) equipped with an electrospray ion source. The Waters Acquity UPLC^TM^ system was equipped with a binary solvent manager, sample manager, column oven, and photodiode array detector. A Waters ACQUITY UPLC HSS T3 analytical column (100 mm × 2.1 mm, 1.8 μm) was used for chromatographic separation of compounds in SC-CO_2_ extract of CTL. The chromatographic parameters were set as follows: column temperature, 40 °C; flow rate, 0.3 mL/min; temperature of the autosampler, 4 °C; mobile phase, solvent A (0.1% formic acid in water) and solvent B (acetonitrile). A linear gradient was applied for elution as follows: 0–1 min, 10–30% B; 1–2 min, 30–50% B; 2–8 min, 50–70% B; 8–13 min, 70–85% B; 13–15 min, 85% B; 15–19 min, 85–10% B; 19–20 min, 10% B. The injection volume of the blank (methanol) and sample was 2 μL. The PDA spectra were obtained by scanning the samples in the range of 190–400 nm.

The mass spectrometric (MS) data were acquired in MS^E^ experiment under sensitivity mode in both positive and negative electrospray ionization (ESI+/−). The acquisition parameters for MS were set as follows: capillary voltage, 2.5 kV; sample cone voltage, 30.0 V; source temperature, 120 °C; desolvation temperature, 450 °C; cone gas flow rate, 50 L/h; desolvation gas flow rate, 900 L/h; source offset, 80 V; acquisition time, 20 min for both polarities. The low-energy collision-induced dissociation (CID) of the MS^E^ experiment was 6 eV, the high-energy CID was 30–85 eV, and the scanning range was *m*/*z* 50–1200. Nitrogen was used as the drying, nebulizing, and collision gas. Leucine enkephalin (200 pg/mL, 5 µL/min) was used as the reference compound in order to obtain exact mass accuracy, with [(M + H)^+^ *m*/*z* 556.2766] as the positive ion and [(M − H)^−^ *m*/*z* 554.2620] as the negative ion. The lock-spray scan time was set at 0.25 s, with an interval of 30 s. The data were acquired and processed by Waters Connect UNIFI version 3.0.0.15.

### 3.5. Chemometric Analysis

For the analysis of qualitative data, the PCA, correlation plots, and hierarchical cluster analysis heatmap diagrams were made with the open-source R software version 3.5.1 by using ggplot2 (https://ggplot2.tidyverse.org/), factoextra (https://cran.r-project.org/web/packages/factoextra/index.html), and ggcorrplot (https://cran.r-project.org/web/packages/ggcorrplot/readme/README.html) packages from the Comprehensive R Archive Network (CRAN) database accessed on 10 June 2023. Venn diagrams were generated using a web tool.

## 4. Conclusions

The present study combined the chromatographic (UPLC-Q-TOF-MS^E^) separation technique with chemometric analysis to establish optimized SC-CO_2_ extraction conditions to achieve maximum fatty acids, fatty amides, and cinnamic acid derivatives from Uttarakhand *C. tamala* leaves. A total of 166 metabolites, of which 118 were fatty acids, 27 fatty amides, and 21 cinnamic acid derivatives, were identified in both positive and negative ion mode, out of which 142 compounds were common and found in all five extracts. This rapid and high-quality chemical analysis revealed that the SC-CO_2_ extraction parameters used in CTL4 were the most optimized in the present study. Moreover, these metabolites possess a lot of interest because of their diverse spectrum of biological functions, especially in the fields of nutraceuticals. To the best of our knowledge, this is the first study to detect the different metabolites in SC-CO_2_ extracts analyzed by UPLC-Q-TOF-MS and justifying the quality of CTL as a flavoring agent and in functional foods.

## Figures and Tables

**Table 1:** Tentative identification of chemical constituents in supercritical-CO_2_ extracts of *C. tamala* leaf using UPLC-Q-TOF-MS^E^ in both positive and negative polarity.

No.	RT	Compound	Chemical Class	Molecular Ion	Observed Mass	Error	MS/MS Fragments	SC-CO_2_ Extracts				
								CTL1	CTL2	CTL3	CTL4	CTL5
1	1.62	Protocatechuic acid	CAD	[M − H]^−^	153.0204	−0.7	109.0297	+	−	−	−	−
2	1.67	3-(4-Hydroxyphenyl)- lactic acid	CAD	[M − H]^−^	181.0496	2.4	119.0502	+	+	+	+	+
3	1.86	Oxodecanedioic acid	DFA	[M − H]^−^	215.0928	−1.4	197.0786 171.1076 155.0751	−	+	+	−	+
4	1.88	Heptanedioic acid (Pimelic acid I)	DFA	[M − H]^−^	159.0667	−2.5	141.0542 115.0772 97.0673	+	+	+	+	−
5	1.90	Salicylic acid	CAD	[M − H]^−^	137.0244	0.0	93.0348	+	+	+	+	+
6	2.16	Heptanedioic acid (Pimelic acid II)	DFA	[M − H]^−^	159.0665	−1.3	141.0542 115.0772 97.0673	+	+	+	+	−
7	2.18	Octanedioic acid (Suberic acid)	DFA	[M − H]^−^	173.082	−0.8	155.0687 129.0986 111.0816	+	+	+	+	+
8	2.21	2-Hydroxyhydro-cinnamic acid	CAD	[M − H]^−^	165.0542	−1.3	119.0502 79.9562	+	+	+	+	+
9	2.22	Hydroxysebacic acid	DFA	[M − H]^−^	217.1095	−6.5	199.0984 171.1049 155.1108	+	+	+	+	+
10	2.32	3-Hydroxy-4-methoxy-cinnamic acid	CAD	[M − H]^−^	193.0517	−5.7	193.0517	+	+	−	+	+
11	2.33	Hydroxyundecanedioic acid	DFA	[M − H]^−^	231.1241	−1.3	213.1229 169.1233	+	+	+	+	+
12	2.40	Syringaldehyde	CAD	[M + H]^+^	183.0653	−0.1	155.0731 123.0470	+	+	+	+	+
13	2.41	Oxododecanedioic acid I	DFA	[M − H]^−^	243.1215	5.4	225.1170 207.1074 181.1243	+	+	+	+	+
14	2.45	Decenedioic acid I	DFA	[M − H]^−^	199.0983	−3.5	181.0865 155.1055 137.0939	+	+	+	+	+
15	2.47	Nonanedioic acid (Azelaic acid)	DFA	[M − H]	187.0982	−3.2	169.0861 143.1065 125.0966	+	+	+	+	+
16	2.50	Oxododecanedioic acid II	DFA	[M − H]^−^	243.1215	5.4	225.1170 207.1074 181.1243	+	+	+	+	+
17	2.60	Oxododecanedioic acid III	DFA	[M − H]^−^	243.1214	5.8	225.1170 207.1074 181.1243	+	+	+	+	+
18	2.65	Dodecenedioic acid I	DFA	[M − H]^−^	227.1301	−5.3	209.1197 183.1368 165.1287	+	+	+	+	+
19	2.65	Decenedioic acid II	DFA	[M − H]^−^	199.0983	−3.5	181.0865 155.1055 137.0939	+	+	+	+	+
20	2.65	Hydroxydodecanedioic acid	DFA	[M − H]^−^	245.1406	−4.9	227.1334 201.1317	+	+	+	+	+
21	2.75	Sebacic acid	DFA	[M − H]^−^	201.113	1.2	183.1021 157.1214 139.1119	+	+	+	+	+
22	2.77	4-Hydroxycinnamic acid	CAD	[M − H]^−^	163.0409	−5.0	119.0495	−	−	+	−	−
23	2.78	4-Methoxycinnamic acid	CAD	[M − H]^−^	177.0556	0.6	133.0653 103.0577 92.0285	+	+	+	+	+
24	2.79	Nonendioic acid	DFA	[M − H]^−^	185.0815	2.2	167.0762, 141.0953, 123.0865	−	−	−	+	−
25	2.79	Salicylic acid	CAD	[M − H]^−^	137.0243	0.7	119.0515 93.0348	+	+	+	+	+
26	2.82	Abscisic acid	CAD	[M − H]^−^	263.1296	−2.7	219.1398 203.1083 153.0899	+	+	+	+	+
27	2.82	*p*-Hydroxybenzoic acid	CAD	[M − H]^−^	137.0249	−3.1	93.0348	+	+	+	+	+
28	2.86	4-Hydroxy cinnamaldehyde	CAD	[M − H]^−^	147.0457	−3.9	119.0481 117.0331	+	+	+	+	+
29	2.92	Undecanedioic acid	DFA	[M − H]^−^	213.1128	1.9	195.1116 169.1233 151.1254	+	+	+	+	+
30	2.93	Decenoic acid	MFA	[M − H]^−^	169.1233	0.6	169.1234 151.1153 125.1298	+	+	+	+	+
31	2.94	Coumarin	CAD	[M + H]^+^	147.0446	0.9	103.0540 91.0597	+	+	+	+	+
32	2.95	Oxodecenoic acid	MFA	[M − H]^−^	183.1028	−1.5	183.1027 147.0874 139.1129	+	+	+	+	+
33	3.04	Decenedioic acid	DFA	[M − H]^−^	215.1292	−1.4	197.1188 171.1410 153.1279	+	+	+	+	+
34	3.06	Cinnamic acid	CAD	[M − H]−	147.0457	0.5	103.0542	+	+	+	+	+
35	3.07	Dodecanedioic acid II	DFA	[M − H]^−^	227.1301	−5.3	209.1197 183.1368 165.1287	+	+	+	+	+
36	3.15	9,10,13-Trihydroxy-11-octadecenoic acid	MFA	[M − H]^−^	329.2325	2.4	311.2269 293.2155 171.1046	+	+	+	+	+
37	3.20	2-Methoxycinnamic acid	CAD	[M − H]^−^	177.056	−1.5	133.0653 103.0577 92.0285	+	+	+	+	+
38	3.21	Cinnamyl alcohol	CAD	[M + H]^+^	135.0851	0.5	117.0695 91.0559	−	−	+	+	−
39	3.33	Dihydroxyhexadecanoic acid	MFA	[M − H]^−^	287.2232	−1.4	269.2183 241.2277	+	+	+	+	+
40	3.39	Dodecanedioic acid	DFA	[M − H]^−^	229.1439	2.9	211.1342 167.1434	+	+	+	+	+
41	3.48	Cinnamaldehyde I	CAD	[M + H]^+^	133.0648	0.9	103.0603 79.0593	+	+	+	+	+
42	3.60	9,10,11-Trihydroxy-12-octadecenoic acid	MFA	[M − H]^−^	329.2325	2.4	311.2269 293.2155 171.1046	+	+	+	+	+
43	3.72	Octadecanedioic acid I	DFA	[M − H]^−^	313.2375	3.2	295.2280 269.2425 251.2289	+	+	+	+	+
44	3.87	Tridecanedioic acid	DFA	[M − H]^−^	243.1601	0.4	225.1506 199.1763 181.1609	+	+	+	+	+
45	4.00	Nonanamide	FAA	[M + H]^+^	158.1559	1.3	116.1119 69.0753	+	+	+	+	+
46	4.00	Methylcinnamic acid	CAD	[M + H]^+^	163.0757	−1.1	105.0356 103.0569 91.0519	+	+	+	+	+
47	4.23	Cinnamyl acetate	CAD	[M + H]^+^	177.0913	−1.2	105.0356 103.0569 91.0519	+	+	+	+	+
48	4.41	Decanamide	FAA	[M + H]^+^	172.1706	−5.8	128.0678 105.0731 69.0751	+	+	+	+	+
49	4.49	Tetradecanedioic acid I	DFA	[M − H]^−^	257.1758	0.1	239.1580 213.1841 195.1700	+	+	+	+	+
50	4.75	Cinnamyl alcohol II	CAD	[M + H]^+^	135.0851	0.5	117.0695 91.0559	+	+	+	+	+
51	4.84	Hexadecanedioic acid	DFA	[M − H]^−^	283.1912	1.1	265.1766 221.1924	+	+	+	+	+
52	4.94	Octadecanedioic acid II	DFA	[M − H]^−^	313.2375	3.2	295.2280 269.2425 251.2289	+	+	+	+	+
53	5.08	Cinnamaldehyde II	CAD	[M + H]^+^	133.0649	0.7	103.0582 77.0431	+	+	+	+	+
54	5.26	Pentadecanedioic acid	DFA	[M − H]^−^	271.1915	0.0	253.1779 227.2038 209.1932	+	+	+	+	+
55	5.40	Octadecanedioic acid I	DFA	[M − H]^−^	311.2224	1.3	293.2123 267.2316 249.2220	+	+	+	+	+
56	5.46	Octadecanedioic acid III	DFA	[M − H]^−^	313.2375	3.2	295.2280 269.2425 251.2289	+	+	+	+	+
57	5.50	Octadecanedioic acid II	DFA	[M − H]^−^	311.2224	1.3	293.2123 267.2316 249.2220	+	+	+	+	+
58	5.53	Heptadecanedioic acid	DFA	[M − H]^−^	297.2067	1.4	279.1973 253.2210 235.2145	+	+	+	+	+
59	5.70	Octadecanedioic acid III	DFA	[M − H]−	311.2224	1.3	293.2123 267.2316 249.2220	+	+	+	+	+
60	5.98	Dihydroxystearic acid	MFA	[M − H]^−^	315.2544	−1.0	315.2544 297.2490	+	+	+	+	+
61	6.03	Hydroxystearidonic acid I	MFA	[M − H]^−^	291.1964	0.7	273.1883 255.2316 245.1916	+	+	+	+	+
62	6.18	Hexadecanedioic acid	DFA	[M − H]^−^	285.2072	−0.35	267.1978 241.2069	+	+	+	+	+
63	6.32	Decanoic acid (Capric acid)	MFA	[M − H]^−^	171.1392	−1.1	171.1396	+	+	+	+	+
64	6.40	Stearidonic acid I	MFA	[M − H]^−^	275.2027	−3.6	257.1952 231.2127 229.1872	+	+	+	+	+
65	6.40	Lauramide	FAA	[M + H]^+^	200.2015	−3.0	116.1121 102.0851 74.0631	+	+	+	+	+
66	6.42	9-Hydroxy-12,14,16-octadecatrienoic acid	MFA	[M − H]^−^	293.2125	−1.0	275.2022 183.1399 171.1017	+	+	+	+	+
67	6.57	Hydroxyoctadecatrienoic acid I	MFA	[M − H]^−^	293.2125	−1.0	275.2076 185.1206 171.1047	+	+	+	+	+
68	6.57	Stearidonic acid II	MFA	[M − H]^−^	275.2027	−3.6	257.1952 229.1872	+	+	+	+	+
69	6.80	Hydroxystearidonic acid II	MFA	[M − H]^−^	291.1964	0.7	273.1883 255.2316 245.1916	+	+	+	+	+
70	6.98	Hydroxystearidonic acid III	MFA	[M − H]^−^	291.1964	0.7	273.1883 255.2316 245.1916	+	+	+	+	+
71	7.16	Hydroxystearidonic acid IV	MFA	[M − H]^−^	291.1964	0.7	273.1883 255.2316 245.1916	+	+	+	+	+
72	7.17	Tridecanamide	FAA	[M + H]^+^	214.2194	0.5	105.0761 91.0597 69.0781	+	+	+	+	+
73	7.22	Heptadecanedioic acid I	DFA	[M − H]^−^	299.2242	−4.7	281.2143 255.2352 237.2166	+	+	+	+	−
74	7.49	13-Hydroxy-9,11-octadecadienoic acid	MFA	[M − H]^−^	295.2278	0.3	277.2161 195.1418 113.0973	+	+	+	+	+
75	7.85	Ricinoleic acid I	MFA	[M − H]^−^	297.2438	−1.0	279.2322 183.1396 93.0349	+	+	+	+	+
76	8.30	Hydroxy- octadecatrienoic acid II	MFA	[M − H]^−^	293.2125	−1.0	257.1911 171.1047	+	+	+	+	+
77	8.33	Octadecanedioic acid IV	DFA	[M − H]^−^	313.2375	3.2	295.2280 269.2425 251.2289	+	+	+	+	+
78	8.50	Hydroxy- octadecatrienoic acid III	MFA	[M − H]^−^	293.2125	−1.0	275.2076 171.1047	+	+	+	+	+
79	8.52	Ricinoleic acid II	MFA	[M − H]^−^	297.2438	−1.0	279.2322 183.1396 93.0349	+	+	+	+	+
80	8.62	Ricinoleic acid III	MFA	[M − H]^−^	297.2438	−1.0	279.2322 183.1396 93.0349	+	+	+	+	+
81	8.84	Dodecanoic acid (Lauric acid)	MFA	[M − H]^−^	199.1704	−0.3	199.1704 181.1572	+	+	+	+	+
82	9.01	Hydroxyhexadecenoic acid I	MFA	[M − H]^−^	269.213	−3.0	251.2080 223.2160	+	+	+	+	+
83	9.11	Palmitoleamide I	FAA	[M + H]^+^	254.2483	−1.8	105.0752 91.0577 69.0753	+	+	+	+	+
84	9.14	Linoleamide	FAA	[M + H]^+^	278.2471	2.7	189.1640 175.1480 91.0578	+	+	+	+	+
85	9.17	Tetradecanedioic acid II	DFA	[M − H]^−^	257.1758	0.1	239.1580 213.1841 195.1700	−	−	+	−	+
86	9.26	9-Hydroxy-10,12-octadecadienoic acid	MFA	[M − H]^−^	295.2278	0.3	277.2229 183.0112 119.0509	+	+	+	+	+
87	9.29	Myristamide	FAA	[M + H]^+^	228.2345	−1.3	116.1097 102.0963 88.0815	+	+	+	+	+
88	9.36	9-Hydroxy-10,12-octadecadienoic acid	MFA	[M − H]^−^	295.2278	0.3	277.2229 183.0112 119.0509	+	+	+	+	+
89	9.51	Nonadecanedioic acid	DFA	[M − H]^−^	327.2549	−2.4	309.2492 283.2639 265.2502	+	+	+	+	+
90	9.81	Hydroxyhexadecenoic acid II	MFA	[M − H]^−^	269.213	−3.0	251.2080 223.2160	+	+	+	+	+
91	9.96	Heptadecanedioic acid II	DFA	[M − H]^−^	299.2242	−4.7	281.2143 255.2352 237.2166	+	+	+	+	+
92	10.14	Dihydroxy- octadecenoic acid	MFA	[M − H]^−^	313.2378	1.9	183.1315 129.0899	+	+	+	+	+
93	10.16	Octadecanedioic acid V	DFA	[M − H]^−^	313.2375	3.2	295.2280 129.0899	+	+	+	+	+
94	10.22	Tridecanoic acid	MFA	[M − H]^−^	213.1856	1.9	213.1856 195.1645	+	+	+	+	+
95	10.27	Hydroxyhexadecenoic acid III	MFA	[M − H]^−^	269.213	−3.0	251.2080 225.2243 223.2160	+	+	+	+	+
96	10.29	Hydroxyhexadecanoic acid I	MFA	[M − H]^−^	271.2293	−5.2	271.2293 225.2244	+	+	+	+	+
97	10.35	Pentadecanamide	FAA	[M + H]^+^	242.2466		116.0578 102. 0954 91.0591	+	+	+	+	+
98	10.50	Dihydroxy- octadecadienoic acid I	MFA	[M − H]^−^	311.2222	1.9	183.1315 129.0899	+	+	+	+	+
99	10.60	Palmitadienoic acid	MFA	[M − H]^−^	251.2016	0.4	251.2016	+	+	+	+	+
100	10.66	Linoleamide I	FAA	[M + H]^+^	280.2631	1.4	88.0805 75.0431 57.0752	+	+	+	+	+
101	10.70	Dihydroxy- octadecadienoic acid II	MFA	[M − H]^−^	311.2222	1.9	293.2160 275.1958 257.2183	+	+	+	+	+
102	10.74	Eicosanedioic acid	DFA	[M − H]^−^	341.2695	0.6	323.2603 297.2877 279.2632	+	+	+	+	+
103	10.77	Nonadecanedioic acid	DFA	[M − H]^−^	325.2368	4.9	307.2291 281.2480 263.2364	+	+	+	−	+
104	11.01	Dihydroxy- octadecadienoic acid III	MFA	[M − H]^−^	311.2222	1.9	293.2160 275.1958 257.2183	+	+	+	+	+
105	11.10	Ceriporic acid I	DFA	[M − H]^−^	351.2534	1.9	333.2467 307.2613 289.2500	+	+	+	+	+
106	11.14	Oleic acid I	MFA	[M − H]^−^	281.248	2.1	281.2481 263.2364 237.2231	−	+	+	+	+
107	11.17	Pentacosanedioic acid I	DFA	[M − H]^−^	411.3474	1.5	393.3307 367.3678 349.3567	+	+	+	+	+
108	11.24	Stearic acid I	MFA	[M − H]^−^	283.2642	0.2	283.2642 265.2568	+	+	+	+	+
109	11.35	Eicosenedioic acid	DFA	[M − H]^−^	339.2542	−0.3	321.2497 295.2707 277.2547	+	+	+	+	+
110	11.37	Hydroxyhexadecanoic acid II	MFA	[M − H]^−^	271.2293	1.5	271.2293 225.2244	+	+	+	+	+
111	11.38	Pentadecenoic acid	MFA	[M − H]^−^	239.2015	0.8	239.2115 221.1918	+	+	+	+	+
112	11.48	Linolenic acid	MFA	[M − H]^−^	277.2173	0.0	259.2143 233.2348 211.1382	+	+	+	+	+
113	11.61	Myristic acid	MFA	[M − H]^−^	227.2015	0.7	227.2015 209.1939	+	+	+	+	+
114	11.80	Oxotetra- cosanedioic acid	DFA	[M − H]^−^	411.3118	−0.5	393.3081 375.2944 349.3106	+	+	+	+	+
115	11.80	Palmitamide	FAA	[M + H]^+^	256.2636	−0.4	116.1119 102.0963 88.0805	+	+	+	+	+
116	11.83	Heptadecadienoic acid	MFA	[M − H]^−^	265.2167	2.3	265.2167 247.2089	+	+	+	+	+
117	11.88	Ceriporic acid II	DFA	[M − H]^−^	351.2534	1.9	333.2467 307.2613 289.2500	−	+	+	+	−
118	11.88	Eicosadienoic acid I	MFA	[M − H]^−^	307.2649	−2.0	289.2500 263.2529 261.2602	−	−	−	+	−
119	11.97	Heneicosanedioic acid	DFA	[M − H]^−^	355.285	1.1	337.2845 311.2908 293.2897	+	+	+	+	+
120	12.10	Ceriporic acid III	DFA	[M − H]^−^	351.2534	1.9	333.2467 307.2613 289.2500	−	−	−	+	−
121	12.25	Palmitoleic acid I	MFA	[M − H]^−^	253.2177	−1.6	253.2177 235.2183	+	+	+	+	+
122	12.49	Ricinoleic acid IV	MFA	[M − H]^−^	297.2438	−1.0	279.2322 183.1396 93.0349	+	+	+	+	+
123	12.51	Oleamide I	FAA	[M + H]^+^	282.2787	1.4	135.1205 83.0896 69.0753	+	+	+	+	+
124	12.52	Arachidamide	FAA	[M + H]^+^	312.3257	1.3	116.0678 102.0963 88.0597	+	+	+	+	+
125	12.70	Pentadecanoic acid	MFA	[M − H]^−^	241.2173	0.0	241.2173 223.2073	+	+	+	+	+
126	12.70	Palmitic acid I	MFA	[M − H]^−^	255.2328	0.6	255.2351 237.2227	+	+	+	+	+
127	12.70	Eicosenoic acid	MFA	[M − H]^−^	309.2783	5.2	309.2799 291.2735	+	−	+	+	+
128	12.85	Heptadecanamide I	FAA	[M + H]^+^	270.2778	4.8	116.0579 88.0597 57.0753	+	+	+	+	+
129	13.04	Linoleic acid	MFA	[M − H]^−^	279.2329	0.4	279.2329 261.2203 243.2081	+	+	+	+	+
130	13.19	Docosanedioic acid	DFA	[M − H]^−^	369.301	0.0	335.3020 325.3030 307.2972	+	+	+	+	+
131	13.22	Heptadecanamide II	FAA	[M + H]^+^	270.2778	4.8	116.0579 88.0597 57.0753	+	+	+	+	+
132	13.33	Palmitoleic acid II	MFA	[M − H]^−^	253.2177	−1.6	253.2177 235.2183	+	+	+	+	+
133	13.40	Arachidinic acid I	MFA	[M − H]^−^	311.295	1.9	311.2950 293.2899 267.2970	+	+	+	+	−
134	13.41	Heptadecenamide	FAA	[M + H]^+^	268.2641	−2.3	116.0579 88.0597 57.0753	+	+	+	+	+
135	13.41	Behenamide I	FAA	[M + H]^+^	340.3575	−0.3	102.0963 88.0431 57.0752	+	+	+	+	+
136	13.48	Palmitoleamide II	FAA	[M + H]^+^	254.2481	−1.0	105.0752 91.0577 69.0753	+	+	+	+	+
137	13.51	Erucamide I	FAA	[M + H]^+^	338.3438	−6.1	321.2128 97.1100 83.0933	+	+	+	+	+
138	13.57	Heptadecenoic acid I	MFA	[M − H]^−^	267.2331	−0.4	267.2331 249.2276	+	+	+	+	+
139	13.66	Palmitoleic acid III	MFA	[M − H]^−^	253.2177	−1.6	253.2177 235.2183	+	+	+	+	+
140	13.70	Palmitic acid II	MFA	[M − H]^−^	255.2328	0.6	255.2351 237.2227	+	+	+	+	+
141	13.77	Heneicosanoic acid	MFA	[M − H]^−^	325.3113	−0.3	325.3113 307.3052 281.3201	+	+	+	+	+
142	13.77	Heptadecanamide III	FAA	[M + H]^+^	270.2778	4.8	115.0579 91.0597 69.0753	+	+	+	+	+
143	13.79	Oleamide II	FAA	[M + H]^+^	282.2789	0.7	69.0753 55.0591	+	+	+	+	+
144	13.82	Heptadecenoic acid II	MFA	[M − H]^−^	267.2331	−0.4	267.2331 249.2276	+	−	+	+	+
145	14.12	Arachidinic acid II	MFA	[M − H]^−^	311.295	1.9	311.2950 293.2899 267.2970	+	+	+	+	+
146	14.30	Palmitic acid III	MFA	[M − H]^−^	255.2328	0.6	255.2351 237.2227	+	+	+	+	+
147	14.37	Heptadecanoic acid I	MFA	[M − H]^−^	269.2482	1.5	269.2482 251.2439 225.2305	+	+	+	+	+
148	14.39	Tricosanedioic acid	DFA	[M − H]^−^	383.3176	−2.4	365.3100 339.3257 321.3157	+	+	+	+	+
149	14.41	Octadecanedioic acid VI	DFA	[M − H]^−^	313.2375	3.19	295.2280 269.2425 251.2289	+	+	+	+	+
150	14.65	Stearamide	FAA	[M + H]^+^	284.2957	−3.2	116.1121 102.0851 88.0821	+	+	+	+	+
151	14.67	Erucamide II	FAA	[M + H]^+^	338.3401	4.9	321.2128 97.1100 83.0933	+	+	+	+	+
152	14.87	Stearic acid II	MFA	[M − H]^−^	283.2642	0.2	283.2642 265.2568	+	+	+	+	+
153	14.87	Ocatdecanoic acid II	MFA	[M − H]^−^	281.2478	2.8	281.2478 263.2364	+	+	+	+	+
154	14.95	Tetracosanoic acid	MFA	[M − H]^−^	367.3573	2.4	367.3573	+	+	+	−	−
155	14.95	Behenamide II	FAA	[M + H]^+^	340.3575	−3.0	102.0963 88.0431 57.0752	+	+	+	+	+
156	15.04	Nonadecanoic acid	MFA	[M − H]^−^	297.2798	0.3	297.2798 279.2667	+	+	+	+	−
157	15.16	Eicosenamide	FAA	[M + H]^+^	310.3092	3.8	256.2669 97.1100 69.0753	+	+	+	+	+
158	15.50	Tricosanoic acid	MFA	[M − H]^−^	353.3405	5.7	353.3405	+	+	−	+	−
159	15.56	Eicosadienoic acid II	MFA	[M − H]−	307.2649	−2.0	289.2500 263.2529 261.2602	+	+	+	+	+
160	15.70	Docosanoic acid (Behenic acid)	MFA	[M − H]^−^	339.3272	−0.9	295.3106 139.0407 119.0496	+	+	+	−	+
161	15.70	Nonadecanamide II	FAA	[M + H]^+^	298.3085	6.4	91.0597 69.0745	+	+	+	+	+
162	15.70	Linoleamide II	FAA	[M + H]^+^	280.2628	2.4	81.0513 69.0745 57.0752	−	+	−	−	−
163	15.81	Tetracosanedioic acid	DFA	[M − H]^−^	397.3307	4.03	379.3195 353.3482 335.3321	+	+	+	+	+
164	15.87	Heptadecanoic acid II (Margaric acid)	MFA	[M − H]^−^	269.2482	1.5	269.2482 251.2439 225.2305	+	+	+	+	+
165	16.07	Henicosanamide	FAA	[M + H]^+^	326.3426	−2.6	91.0597 69.0753	−	+	−	+	+
166	16.23	Pentacosanedioic acid II	DFA	[M − H]^−^	411.3474	1.16	393.3307 367.3678 349.3567	+	−	+	+	+

(CTL1) 100 bar/55 °C; (CTL2) 15 0 bar/55 °C; (CTL3) 250 bar/55 °C; (CTL4) 300 bar/55 °C; (CTL5) 500 bar/55 °C; monocarboxylic fatty acid (MFA); dicarboxylic fatty acid (DFA); fatty acid amide (FAA); cinnamic acid derivative (CAD); I–VI indicates presence of isomers; (a) identification based on mass spectrometry data and comparison with the online database with the reference standards; (+)/(–) indicates presence/absence of compound in corresponding extract.

## Data Availability

Data are contained within the article.
